# The New Runner's High? Examining Relationships Between Cannabis Use and Exercise Behavior in States With Legalized Cannabis

**DOI:** 10.3389/fpubh.2019.00099

**Published:** 2019-04-30

**Authors:** Sophie L. YorkWilliams, Charleen J. Gust, Raeghan Mueller, L. Cinnamon Bidwell, Kent E. Hutchison, Arielle S. Gillman, Angela D. Bryan

**Affiliations:** ^1^Department of Psychology & Neuroscience, University of Colorado Boulder, Boulder, CO, United States; ^2^Institute of Cognitive Science, University of Colorado Boulder, Boulder, CO, United States

**Keywords:** cannabis, marijuana, exercise, cannabis legalization, health

## Abstract

Scientific literature examining cannabis use in the context of health behaviors, such as exercise engagement, is extremely sparse and has yielded inconsistent findings. This issue is becoming increasingly relevant as cannabis legalization continues, a situation that has been associated with increased initiation of use among adults, and increased potency of available products in legalized states. Physical activity is among the most important health behaviors, but many Americans do not meet minimum exercise recommendations for healthy living. Common issues surrounding low exercise rates include inadequate enjoyment of and motivation to exercise, and poor recovery from exercise. It is unclear whether cannabis use shortly before and/or after exercise impacts these issues, and whether this co-use affects exercise performance. The present online survey study examines attitudes and behaviors regarding cannabis use with exercise among adult cannabis users living in states with full legal access (*N* = 605). Results indicated that the majority (81.7%) of participants endorsed using cannabis concurrently with exercise, and those who did tended to be younger and more likely to be males (*p* < 0.0005 for both). Even after controlling for these differences, co-users reported engaging in more minutes of aerobic and anaerobic exercise per week (*p* < 0.01 and *p* < 0.05, respectively). In addition, the majority of participants who endorsed using cannabis shortly before/after exercise reported that doing so enhances their enjoyment of and recovery from exercise, and approximately half reported that it increases their motivation to exercise. This study represents an important step in clarifying cannabis use with exercise among adult users in states with legal cannabis markets, and provides guidance for future research directions.

## Introduction

As evidenced by the increasing legalization of both medical and recreational cannabis in the United States (1), public acceptance of cannabis use is growing. Despite this, there is a relative lack of literature exploring the public health implications of cannabis consumption. One health behavior that may be impacted by increased cannabis use is exercise. The benefits of regular exercise are far-reaching and include the prevention or improvement of some cancers, cardiovascular disease, diabetes, depression, Alzheimer's disease, and memory loss (2–4). As a reflection of these benefits, the American College of Sports Medicine (ACSM) recommends that adults participate in at least 150 min of moderate- to vigorous-intensity exercise each week (5). Unfortunately, self-report data indicate that fewer than 50% of US adults meet these minimum recommendations (6). These low rates of exercise participation are indicative of a significant and urgent health concern. Given popular stereotypes that cannabis use is associated with a lack of motivation and, indeed, extreme sedentary behavior (c.f., “couch lock”), increased cannabis use could worsen the sedentary lifestyle epidemic that the US is currently facing. On the other hand, cannabis use is banned by the World Anti-Doping Agency because of its potential to *improve* sports performance, and numerous reports in the popular press suggest that endurance athletes utilize cannabis in their training [e.g., Dreier (7)]. Such conflicting ideas about the relationship between cannabis use and physical activity necessitate further investigation into how cannabis use may affect factors associated with regular physical activity participation.

The sparse empirical literature base that speaks to the association between cannabis and health emphasizes potential negative and harmful effects associated with use, such as psychosis, impaired driving, and sleep disturbances (8–10). There is also an established relationship between cannabis use and increased caloric intake (11), which is especially worrisome given the current obesity epidemic; the Centers for Disease Control and Prevention reported in 2017 that nearly 40% of the US population is obese (12). However, recent results from representative national surveys suggest a lower prevalence of obesity in cannabis users than in non-users (13). Further, cross-sectional data from the National Center for Health Statistics indicate positive correlations with cannabis use and measures of metabolic health, such as lower levels of fasting insulin, smaller waist circumference, and reduced prevalence of type II diabetes (14). There is also evidence to suggest that cannabis use is associated with a greater likelihood of meeting the aforementioned exercise guidelines (15), possibly due to positive influences on motivation or reductions in inflammation and pain during and after exercise (16).

Common barriers that prevent individuals from the initiation or continuation of a regular exercise regimen include (1) improper recovery after exercise (17), (2) lack of motivation (18), and (3) low enjoyment of exercise (19). Cannabis might interact with these barriers in contradictory ways, both positively (e.g., by promoting recovery from or enjoyment of exercise) and negatively (e.g., through decreases in motivation). Gillman et al. (16) address this notion in a review paper commenting on the biological and neurocognitive mechanisms by which cannabis use might impact three potential areas of influence: the effect of cannabis on exercise performance, motivation for exercise, and recovery from exercise. For example, Gillman et al. (16) report that few empirical studies have examined the relationship between cannabis use and exercise performance, and those that have are limited in their external validity due to the use of uncommon forms of cannabis for consumption (e.g., cannabis cigarettes containing unusually low-THC strains). Further, while anecdotal evidence suggests that cannabis reduces motivation for exercise, several neurobiological mechanisms connected to the endocannabinoid system argue against this; rather, there is evidence to suggest that cannabis may have beneficial effects on exercise motivation (16). Finally, while there is evidence that cannabis reduces pain and inflammation in humans (20), other research suggests the use of anti-inflammatory agents, such as cannabinoids, might actually interfere with proper recovery from exercise (16). These limitations and inconsistencies point to a clear need for additional investigations of the effects of cannabis use on health behaviors generally and on exercise in particular (16). Such research is particularly pressing in states with full legal cannabis access, as cannabis use is on the rise in these states and may include broader populations than have been represented in research conducted when cannabis use was an illegal behavior (21).

The present study aimed to address research questions set out by Gillman et al.'s review, and is the first to collect empirical data on attitudes and behaviors regarding cannabis use and exercise among current cannabis users living in states with full legal access. In particular, we were interested in examining differences between users who consume cannabis shortly before or after exercise (co-use), compared to those who do not. Building on Gillman et al. (16), we were also interested in attitudes regarding the impact of cannabis use before or after exercise on exercise performance, enjoyment, motivation, and recovery. Data were gathered using a web-based survey advertised to users in states with legal recreational cannabis. Since endorsement of the use of cannabis concurrent with exercise inherently increases the likelihood of exercise engagement (i.e., a cannabis user who does not exercise cannot use cannabis concurrent with exercise), we hypothesized that co-users would report more exercise behavior than non-co-users. Based on data supporting the anti-inflammatory and analgesic effects of cannabis (16), we hypothesized that users of cannabis concurrent with exercise would report that it aided in recovery. Finally, given conflicting findings regarding performance and motivation, and little to no data on enjoyment, we considered tests of these associations to be exploratory.

## Methods

### Procedures

A voluntary, anonymous online “Cannabis and Health” survey was advertised on Facebook and targeted individuals aged 21 and older living in California, Colorado, Nevada, Oregon, and Washington and who “liked” pages related to cannabis use (e.g., Cannabis Culture Magazine). In Colorado, the survey was also advertised through medical cannabis card registration clinics and dispensaries in the Boulder-Denver area. The survey was hosted on Qualtrics.com and data were collected from January 2017 to July 2018. Survey details are described in another recent publication (22). Participants were asked demographic questions, along with health, exercise, and substance use questions. They were free to skip questions, other than acknowledgment of consent and that they were 21 years of age or older. Inclusion criteria for the current study were as follows: (1) reported living in a state with legal recreational cannabis, and (2) responded to the cannabis and exercise questions (details below).

In the initial iteration of the survey, we asked, “*Do you ever use cannabis within 1 hour before and/or 4 hours after you engage in exercise?*” This version was completed by 260 (43.0%) of the present study participants. Subsequently, in an effort to delineate those who use cannabis either before or after exercise, the survey was altered and the remaining participants (*n* = 345; 57.0% of present study) were asked (1) “*Do you ever use cannabis within 1 hour before you engage in exercise?*” and (2) “*Do you ever use cannabis within 4 hours after you engage in exercise?*” Participants who answered “yes” to the single question in the first version, or “yes” to either question in the latter version, were categorized as individuals who use cannabis concurrent with exercise for the full-sample analyses.

Participants who endorsed cannabis use concurrent with exercise were given the following instructions. “The following statements are examples of why you might use cannabis before or after exercise. Please indicate how much you agree with each statement.” They were presented with cannabis-exercise attitude statements (listed below) and asked to indicate their opinion of each on a 7-point likert scale, with the anchors of 1 = “strongly disagree,” 4 = “neither agree nor disagree,” and 7 = “strongly agree.” Statements started with “I believe that” and ended with (1). cannabis enhances my exercise performance; (2). cannabis increases my enjoyment of exercise; (3). I am more motivated to engage in exercise when I use cannabis; (4). cannabis makes it easier to recover from my exercise experience (e.g., reduces inflammation, joint pain, etc.).

### Analyses

RStudio software was utilized in all analyses. We first computed basic demographic information to describe the study sample, and calculated frequencies of concurrent exercise-cannabis use endorsement in the study. Group differences between those who endorsed cannabis use concurrent with exercise and those who did not were examined with χ^2^ tests or *t*-tests as appropriate, for age, gender, ethnicity, body mass index (BMI), state of residency, and frequency of cannabis use. Next, we used *t*-tests to examine differences between co-using and non-co-using groups on minutes of aerobic and anaerobic exercise per week. When group differences arose, we verified that they were not explained by demographic differences, by including demographic variables that were different between groups as covariates in linear regression models.

Next, we used two methods to examine cannabis-exercise attitudes among participants who endorsed co-use. First we examined attitudes numerically, by averaging scores for each of the 4 attitudes. We did this in the full co-using sample, and then examined whether average attitudes differed by type of co-use endorsement (cannabis use *only* before, *only* after, or *both* before and after) using between-subjects ANOVAs. This temporal specificity was only available in the 2-question version of the study, so participants from the 1-question version were included in the model as one group, and those from the 2-question version were grouped by type of co-use endorsement. Second, in the full sample of concurrent users, we categorized interpretations of these attitudes by percentage of participants who agreed or disagreed with each statement. A rating between 1 and 3 was categorized as “disagree,” 4 as “neutral,” and 5–7 as “agree.”

## Results

### Study Demographics

Among the 620 participants that completed either version of the survey and reported residency in a state with legal cannabis, 605 respondents provided answers to the cannabis-exercise questions and were included in the present study. Mean age was 37.5 (SD = 15.7; min = 21; max = 78), and 281 (46.6%) were female. State of residency reports were: 419 (69.3%) in Colorado, 99 (16.4%) in California, 38 (6.3%) in Oregon, 36 (6.0%) in Washington, and 13 (2.1%) in Nevada. The majority (78.3%) of participants were white non-Hispanic, followed by 10.6% Hispanic, 4.0% Native American, 3.8% black or African American, 1.5% Asian, 0.05% Pacific Islander, and 1.3% declined to answer.

### Cannabis Use Before/After Exercise

In the full sample, 494 participants (81.7%) endorsed using cannabis before and/or after exercise and 111 (18.3%) did not. Among the sample from the first version of the survey, who answered the single cannabis-exercise question (*n* = 260), 79.6% endorsed using cannabis before and/or after exercise (*n* = 207), and 20.4% reported that they did not (*n* = 53). Among the 345 participants in the 2-question sample, 287 (83.3%) endorsed at least one of the two cannabis-exercise questions. Specifically, 237 (68.9%) endorsed using cannabis within 1 h *before* exercise, while 282 (82.0%) endorsed using cannabis within 4 h *after* exercise, and 58 (16.8%) reported that they did not use cannabis within 1 h before nor within 4 h after engaging in exercise. Interestingly, 232 of the 345 2-question sample participants endorsed using cannabis *both* before and after exercise (67.2%), while 50 (14.5%) endorsed only using after exercise, and only 5 (1.5%) endorsed only using before exercise. Thus, most cannabis users who use cannabis concurrent with exercise report doing so both before and after exercise.

### Demographic and Lifestyle Differences Between Co-users and Non-co-users

Bivariate relationships suggested that participants who used cannabis with exercise were younger, more likely to be male, and of lower BMI than those who did not use cannabis with exercise. Average age of participants who used cannabis with exercise was 36.3 (SD = 14.9), while the average age of cannabis users who did not was 43.1 (SD = 18.0; *p* < 0.0005; *d* = −0.39). Females comprised only 42.8% of participants who endorsed using cannabis with exercise, compared to 63.1% of those who did not *p* < 0.0005). Average reported BMI of co-using participants was 25.6 (SD = 5.61), while the average was 27.0 (SD = 6.0; *p* < 0.05; *d* = −0.24) among those who did not use cannabis with exercise. However, these group differences in reported BMI were not present after age and gender, which were different between co-users and non-co-users, were accounted for. Participant race/ethnicity and state of residency did not differ between exercise groups.

Co-users endorsed using cannabis flower 5.5 days per week on average (SD = 2.5; min = 0; max = 7), while non-co-users endorsed using 3.2 days (SD = 3.1; min = 0; max = 7; *p* < 0.0001; *d* = 0.76). Co-users endorsed using high-potency cannabis concentrate 2.5 days per week on average (SD = 2.9; min = 0; max = 7), while non-co-users endorsed using 0.6 days (SD = 1.6; min = 0; max = 7; *p* < 0.0001; *d* = 1.0). Co-users endorsed using cannabis edibles 1.3 days per week on average (SD = 2.0; min = 0; max = 7), while non-co-users endorsed using 1.6 days (SD = 2.4; min = 0; max = 7; *ns*). Even after controlling for age and gender, co-users used cannabis flower on 2.4 more days (*p* < 0.0001) and cannabis concentrates 1.8 more days (*p* < 0.0001) than non-co-users on average.

### Differences in Exercise Behaviors Between Co-users and Non-co-users

Co-using participants reported 159.7 min per week of aerobic exercise on average (SD = 154.3; min = 0; max = 600), while non-co-users reported an average of 103.5 min (SD = 113.6; min = 0; max = 510; *p* < 0.0001; *d* = 0.45). Using linear regression to control for potential demographic confounds of age and gender, use of cannabis with exercise was still associated with 43.4 more min of weekly aerobic exercise on average (*p* < 0.01). Consistent with this finding, 40.1% of cannabis users who used with exercise met or exceeded American College of Sports Medicine's recommendations of a minimum of 150 min of aerobic exercise per week (5), compared to only 28.7% of cannabis users who did not endorse using with exercise (*p* = 0.036). This discrepancy was not limited to aerobic activity. Cannabis users who used cannabis during exercise also reported an average of 37.4 more minutes of anaerobic exercise (mean = 101.6; SD = 129.1; min = 0; max = 600) than cannabis users who did not use during exercise (mean = 64.2; SD = 104.3; min = 0; max = 600; *p* < 0.005; *d* = 0.33). After controlling for the demographic variables that were different between groups (age and gender), cannabis use during exercise was still associated with 30.2 more minutes of reported anaerobic exercise (*p* = 0.033).

### Attitudes About Cannabis Use Before/After Exercise

Mean scores on the 1–7 scales for each cannabis-exercise attitude statement among all participants who endorsed co-use were as follows: performance was 4.4 (SD = 1.6; median = 4), enjoyment was 5.3 (SD = 1.6; median = 6), motivation was 4.7 (SD = 1.8; median = 5), and recovery was 5.7 (SD = 1.5; median = 6). With a score of 4 equating to neither agreement nor disagreement, and 7 equating to strong agreement, results indicate that average attitudes, particularly toward enjoyment and recovery, were above neutral when the full co-using sample was combined (Table 1). Of note, more respondents endorsed strong agreement than strong disagreement with the attitude statements (i.e., there were more answers of 6 and 7 than 1 and 2). As such, the median results may represent participant attitudes better than the means and standard deviations. To add nuance to these attitudes, we wanted to examine whether they differed across participants who endorsed cannabis use *only* before, *only* after, or *both* before and after exercise. This level of detail was only available in the 2-question version of the study, so participants from the 1-question version were treated as one group. Due to the low number of survey participants who endorsed cannabis use *only* before exercise in the second version of the survey (*n* = 5), they were not included in the ANOVA models. Thus, the 3 groups in the model were: those in the second survey who endorsed cannabis use *both* before and after exercise, those in the second survey who endorsed cannabis use *only* after exercise, and those in the first version of the survey who endorsed our single-question regarding cannabis use before or after exercise. Average scores for each attitude statement by group are detailed in Table 2. We found differences in subjective experience of those who reported cannabis use *only* after exercise, compared to those who used *both* before and after, and those who participated in the combined single-question survey version (*p* < 0.0001 for all attitudes). This difference was most prominent for enjoyment, motivation, and performance.

**Table 1:** Attitudes on how use of cannabis with exercise impacts exercise performance, enjoyment, motivation, and recovery, among all participants who endorsed co-use (*N* = 494).

	**Mean**	**SD**	**Median**
Performance	4.4	1.6	4
Enjoyment	5.3	1.6	6
Motivation	4.7	1.8	5
Recovery	5.7	1.5	6

*Rating options range from 1 (“strongly disagree) to 7 (“strongly agree”)*.

**Table 2:** Attitudes on how use of cannabis with exercise impacts exercise performance, enjoyment, motivation, and recovery, among participants who endorsed co-use.

			**Performance**		**Enjoyment**		**Motivation**		**Recovery**	
**Version**	**Group**	**n**	**Mean**	**SD**	**Mean**	**SD**	**Mean**	**SD**	**Mean**	**SD**
2-question	Before & after	230	4.7	1.5	5.5	1.4	5.0	1.7	5.8	1.3
	Only after	50	2.8^***^	1.3	3.2^***^	1.6	2.9^***^	1.5	5.2^***^	1.8
1-question	Co-users	206	4.4	1.5	5.5	1.6	4.7	1.8	5.6	1.5

*In the 2-question survey version, groups are further delineated by endorsement of use both before and after exercise, or only after exercise. Rating options range from 1 (“strongly disagree) to 7 (“strongly agree”)*.

*** *Group mean comparison p < 0.0001*.

**Figure 1:**
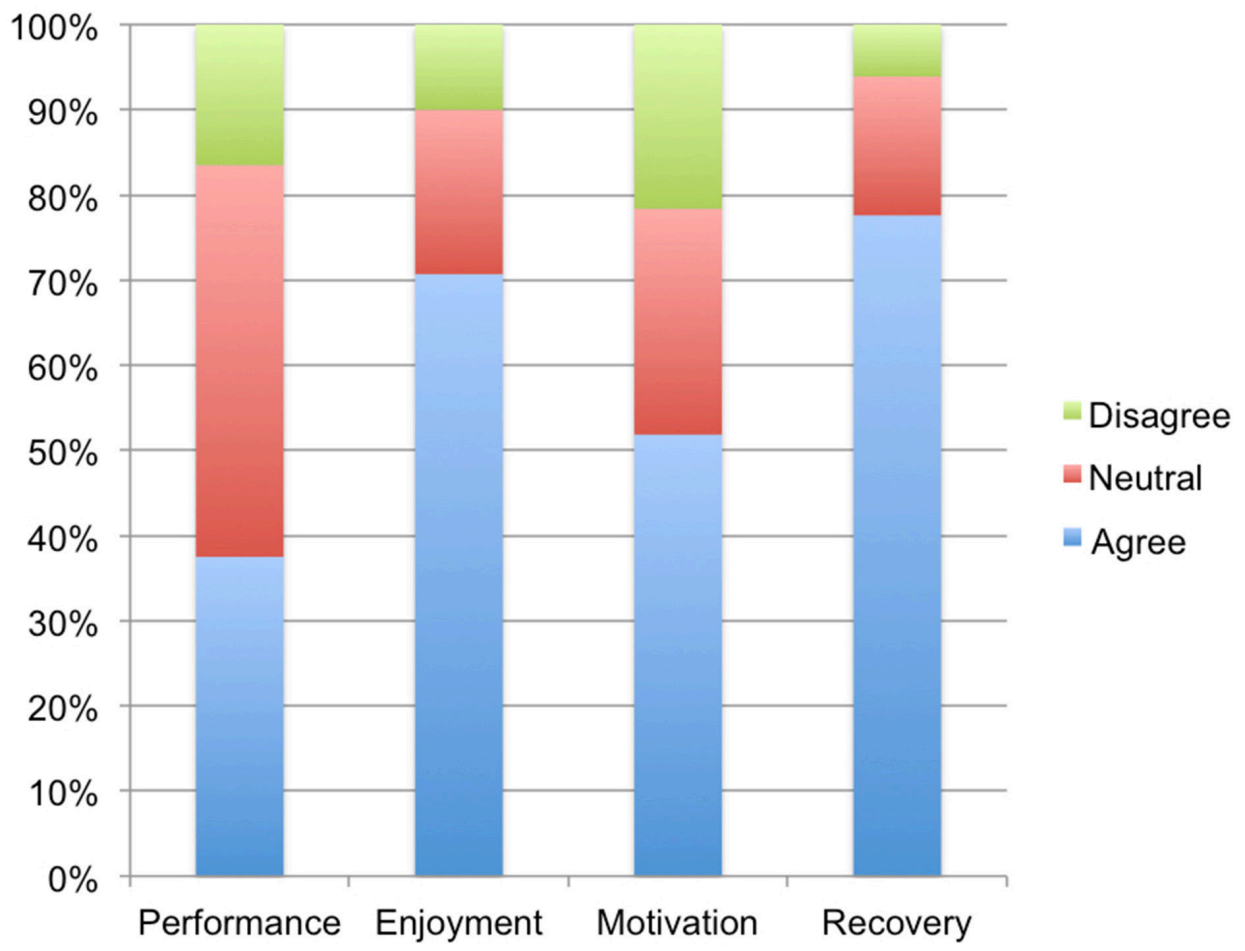
Percentage of co-using participants who agreed, disagreed, or felt neutral toward whether use of cannabis shortly before and/or after exercise enhanced exercise performance, enjoyment, motivation, and recovery.

In order to characterize these attitudes more broadly in all participants who endorsed use before and/or after exercise, we transformed these numeric ratings into categories, grouping all ratings below 4 (“neither agree nor disagree”) as “disagree,” and all ratings above 4 as “agree.” Figure 1 provides a visual representation of these attitudes data. Among participants who endorsed using cannabis before and/or after exercise (*n* = 494), the majority (70.7%) agreed or agreed strongly that cannabis increases enjoyment of exercise, 19.3% were neutral, and 10.0% disagreed or disagreed strongly. The majority (77.6%) also agreed or agreed strongly that cannabis enhances recovery from exercise, while 16.3% were neutral and 6.1% disagreed or disagreed strongly. In contrast, just over half (51.8%) agreed or agreed strongly that cannabis increases motivation to exercise, 26.5% were neutral, and 21.6% disagreed or disagreed strongly. Finally, a minority (37.5%) agreed or strongly agreed that cannabis enhances exercise performance, while almost half (46.0%) were neutral and 16.5% disagreed or disagreed strongly.

## Discussion

The current study aimed to characterize differences between adult cannabis users who consume cannabis shortly before and/or after exercising and those who do not, in the context of legal cannabis markets in the United States. Combining across survey versions of cannabis users, we found that the majority of our sample did endorse using cannabis concurrently with exercise. These participants were younger on average, and included a greater proportion of males than those who do not (both *p* < 0.0005). In line with our hypotheses, they also reported more minutes of aerobic and anaerobic exercise per week (*p* < 0.01 and *p* < 0.05, respectively), even after accounting for potential demographic confounds of age and gender. Concurrent users reported consuming cannabis flower and concentrates more often than the non-co-using group (both *p* < 0.0001, even after controlling for age and gender), but edible use did not differ between groups. Reasons for these correlations can only be speculated upon. It may be that more frequent cannabis users are more likely to engage in co-use simply by virtue of them using so often, rather than reasons related to exercise performance, enjoyment, motivation, or recovery.

Additionally, the present study sought to take initial steps in understanding possible mechanisms through which cannabis could influence exercise behavior, by examining attitudes toward how cannabis co-use influences the exercise experience. We found that the majority of participants who endorsed using cannabis concurrently with exercise reported that doing so at least somewhat enhances recovery from and enjoyment of exercise, while approximately half reported that it at least somewhat increases motivation, and a minority reported that it enhances performance. These findings supported our hypothesis that co-users may be co-using because they believe it contributes to recovery after exercise. The findings also suggest that co-use may facilitate enjoyment of exercise, and (for a subset of co-users) motivation to exercise. Given that these are recognized barriers to exercise (17–19), it is possible that cannabis might actually serve as a benefit to exercise engagement. Finally, the attitudes toward co-use and performance in our participants seem to concur with studies suggesting that cannabis use does *not* enhance exercise performance for most users (16).

To our knowledge, this is the first study to survey attitudes and behavior regarding the use of cannabis before and after exercise, and to examine differences between cannabis users who engage in co-use, compared to those who do not. Given both the spreading legalization of cannabis and the low rates of physical activity in the US, it behooves public health officials to understand the potential effects—both beneficial and harmful—of cannabis use on exercise behaviors (16). Cannabis use has become more common in the past decade, especially among adults in states with legal recreational cannabis (21). As legal risks associated with cannabis use decrease, it is plausible that a broader range of individuals will be initiating use. Thus, the present study's focus on legal market populations is timely, and results may help to anticipate the future landscape of other states, as legalization progresses.

Our results suggest that prior findings of cannabis users being more likely to meet official exercise recommendations (15) may be at least partly associated with perceived impacts of cannabis co-use on enjoyment, reductions in pain and inflammation during and after exercise, and to a lesser extent motivation. Furthermore, participants who use cannabis before and/or after exercise reported that they exercised more, and had positive attitudes about co-use on exercise, which implies cannabis may be a useful tool for exercise among some users. In other words, sedentary cannabis users, particularly those who attribute low physical activity to concerns about recovery, motivation, or enjoyment, may benefit from co-use, provided that they select low-risk exercise options that do not compromise safety during intoxication. However, since the present study did not query potential negative consequences of co-use, any recommendations are premature. For instance, the 16.5% of co-users who reportedly disagreed that co-use enhanced performance may exercise at a lower intensity when they use cannabis shortly beforehand. Similarly, the 6.1% who disagreed that co-use aided in recovery may have experienced injury from exercising while intoxicated. Clearly, more nuanced investigation is needed. Given the diversity of cannabis users in legal states (22), co-use may have a net-positive impact on some, and a net-negative impact for others. Potency and amount of cannabis used, type and context of exercise, and individual health status likely impact whether co-use is a safe and beneficial option.

Limitations of the current study include its cross-sectional, anonymous, and self-report nature, as well as recruitment issues, particularly that of cannabis users without a non-using comparison group. It is possible that recruitment through social media captured respondents who are invested in promoting cannabis, leading to results that are skewed favorably toward exercise. However, it is also possible that recruitment through medical cannabis card registration clinics might have captured respondents who are less physically able, thus leading to results skewed against exercise. It is also critical to recognize that there is substantial overlap between states that have legalized cannabis and states that have higher levels of physical activity. For whatever historical, political, or economic reasons, it is the case that states with legal access to cannabis are, without exception, more physically active than the national average (23). Without a non-using comparison group, we are unable to rule out potential selection bias; that is, it might be that non-users living in such highly active states are even more active than cannabis users. In addition, our findings about exercise behaviors and attitudes regarding cannabis co-use with exercise may not be representative of states with different cannabis legality and may not translate to future behaviors and attitudes if and when states with lower rates of physical activity legalize cannabis. However, given the positive associations that co-users reportedly experience between cannabis use and exercise enjoyment and recovery, there may indeed even be a *stronger* correlation between using cannabis with exercise and exercise behaviors in less active states. This will be a fascinating research question as more states move toward legalization. It is important to note that the current study represents a first attempt to elucidate this complex issue, and that there are currently no grounds for suggestion that non-users should initiate cannabis use to increase their physical activity participation.

As discussed above, future research is needed to refine and expand upon this foundational study. For instance, querying both users and non-users in legal states would clarify the extent to which cannabis use impacts exercise behaviors in these regions. For example, it could be that the non-cannabis users in these highly physically active states are even more active than cannabis users. We are unable to directly test this question with our survey data. Additionally, while the current study illuminates attitudes regarding co-use, it would be useful to explore how often cannabis is used specifically to achieve exercise goals and in what contexts (e.g., specific forms of exercise and during group vs. individual exercise). It is particularly important to explore potential negative outcomes of cannabis co-use with exercise in future research, especially regarding accident or injury as a result of intoxication. Additionally, a longitudinal observational study design could be used to examine how changes in cannabinoid content and product potencies influence exercise behavior. Since we are unable to randomly assign sedentary individuals to use cannabis (or not) and subsequently observe exercise behavior, and because demographic differences can impact exercise engagement, propensity score matching could be used to estimate the effect cannabis legalization has on exercise behavior by equating co-users and non-co-users on influential covariates. Lastly, a useful tool to examine the influence of cannabis on exercise engagement at the population level would be to implement an interrupted time-series study design where exercise behavior is surveyed in a state before and after cannabis legalization.

In summary, these data suggest that many cannabis users in states with legal cannabis access use in conjunction with exercise, and that most who do so believe it increases enjoyment of, recovery from, and to some extent the motivation to engage in exercise. As these factors positively correlate with exercise behavior (24), using cannabis with exercise may play a beneficial role in the health of cannabis users. Recommendations around cannabis use with exercise are premature until aforementioned research questions have been addressed, including independent verification of self-reported behaviors and negative consequences of co-use.

## Ethics Statement

This study was carried out in accordance with the recommendations of the University of Colorado Boulder's Institutional Review Board. Informed consent was given by all participants in accordance with the Declaration of Helsinki and all procedures were approved by the University of Colorado Boulder's Institutional Review Board.

## Author Contributions

KH, LB, SY, and RM contributed to the design and implementation of the research. SY managed and analyzed the data. SY, AB, RM, and LB contributed to the interpretation of the results. SY, RM, and CG wrote the manuscript. SY, AB, CG, and LB edited the manuscript. All authors provided critical feedback and helped shape the manuscript.

### Conflict of Interest Statement

The authors declare that the research was conducted in the absence of any commercial or financial relationships that could be construed as a potential conflict of interest.
